# Match me if you can: Evidence for a domain-general visual comparison ability

**DOI:** 10.3758/s13423-021-02044-2

**Published:** 2022-01-07

**Authors:** Bethany Growns, James D. Dunn, Erwin J. A. T. Mattijssen, Adele Quigley-McBride, Alice Towler

**Affiliations:** 1 College of Social Sciences and International Studies, University of Exeter, Exeter, UK; 2 School of Social and Behavioural Sciences, Arizona State University, Phoenix, AZ USA; 3 School of Psychology, University of New South Wales, Sydney, NSW 2052 Australia; 4 Behavioural Science Institute, Radboud University Nijmegen & The Netherlands Forensic Institute, Nijmegen, the Netherlands; 5 Wilson Center for Science and Justice, Duke University, Durham, NC USA

**Keywords:** Individual differences, Visual comparison, Perceptual expertise, Forensic science

## Abstract

Visual comparison—comparing visual stimuli (e.g., fingerprints) side by side and determining whether they originate from the same or different source (i.e., “match”)—is a complex discrimination task involving many cognitive and perceptual processes. Despite the real-world consequences of this task, which is often conducted by forensic scientists, little is understood about the psychological processes underpinning this ability. There are substantial individual differences in visual comparison accuracy amongst both professionals and novices. The source of this variation is unknown, but may reflect a domain-general and naturally varying perceptual ability. Here, we investigate this by comparing individual differences (*N* = 248 across two studies) in four visual comparison domains: faces, fingerprints, firearms, and artificial prints. Accuracy on all comparison tasks was significantly correlated and accounted for a substantial portion of variance (e.g., 42% in Exp. 1) in performance across all tasks. Importantly, this relationship cannot be attributed to participants’ intrinsic motivation or skill in other visual-perceptual tasks (visual search and visual statistical learning). This paper provides novel evidence of a reliable, domain-general visual comparison ability.

People complete many complex visual tasks in their day-to-day life. One such task is visual comparison—comparing visual stimuli shown side by side and providing judgements about whether they originate from the same or different origins (i.e., “match”). This complex task involves many cognitive and perceptual processes—including visual perception, memory, similarity judgements, categorization and decision-making (Busey & Dror, 2011; Growns & Martire, 2020b), and is used in important real-world judgements. For example, forensic science examiners in feature-comparison disciplines “match” evidence samples (e.g., firearms, faces, fingerprints) to provide judgments about the source of the evidence to investigators or in court (Towler et al., 2018). Critically, it is *human* decision-makers who complete this task with limited input from technology (Thompson et al., 2013; Towler, Kemp, & White, 2017)—making it vital to understand how individuals perform these tasks. Yet research is only beginning to explore human performance in visual comparison.

Professional examiners typically outperform novices on tasks within their domain of experience: facial examiners outperform novices on facial comparison (Phillips et al., 2018; Towler, White, & Kemp, 2017; White, Phillips, et al., 2015; White et al., 2020); fingerprint examiners outperform novices on fingerprint comparison (Busey & Vanderkolk, 2005; Tangen et al., 2011; Ulery et al., 2011); firearm examiners have a higher rate of correct matches than do standard computer algorithms in firearm comparison (Mattijssen et al., 2021); and document examiners are better at avoiding the errors that novices make in handwriting comparison (Bird, Found, Ballantyne, & Rogers, 2010; Bird, Found, & Rogers, 2010; Kam et al., 1997). This superior visual comparison performance is typically attributed to the acquisition of domain-*specific* knowledge within an examiners’ domain of expertise—that is, examiners’ skill is attributed to their training and experience. For example, fingerprint and document examiners have better knowledge of statistical frequencies in forensic stimuli within their domain of expertise (Growns et al., 2021; Martire et al., 2018; Mattijssen et al., 2020), but not outside their domain (Growns & Martire, 2020a, 2020b). Further, fingerprint examiners’ also outperform novices in visual search tasks with fingerprints, but do not outperform novices in the same task with nonfingerprint stimuli (Searston & Tangen, 2017a). The domain-specific nature of examiners’ skill could be unsurprising given that cognitive psychology typically attributes superior performance and expertise to deliberate practice and experience engaging in a task (Charness et al., 2005; Ericsson, 2007, 2014).

Yet something else may be at play in accurate visual comparison performance beyond simply experience or deliberate practice—something that is hinted at by individual differences in this task. While forensic examiners outperform novices as a group, there is substantial variation in visual comparison accuracy even among professionals with equivalent training and experience (Busey & Vanderkolk, 2005; Mattijssen et al., 2020; Phillips et al., 2018; Searston & Tangen, 2017b). Further, facial examiners’ accuracy does not increase with their length of employment (White, Dunn, et al., 2015), and individual differences in fingerprint trainees’ skills are maintained even after 12 months of training (Searston & Tangen, 2017b). This variation in visual comparison ability suggests other factors may contribute to accurate performance beyond experience, deliberate practice, or training.

Recent evidence suggests individual differences in visual comparison could also be driven, at least in part, by a domain-*general* comparison ability. People with superior face *recognition* skills—or the ability to identify faces (“super-recognizers”; Noyes et al., 2017; Russell et al., 2009), also score above average on primate-face and fingerprint-comparison tasks (Towler, Dunn, et al., 2021a). Further, fingerprint examiners not only outperform novices in fingerprint-comparison (i.e., domain-specific; Busey & Vanderkolk, 2005; Tangen et al., 2011), but also on face-comparison tasks (i.e., domain-general; Phillips et al., 2018). Together, this emerging evidence suggests visual comparison may be driven by both a domain-specific skill and a natural domain-*general* visual comparison skill.

Overall, this converging evidence provides a first hint that there may be a generalizable visual comparison ability in specialist populations. However, no research has investigated this in the general population to determine whether it is a domain-general and naturally varying ability. Similar variable and domain-general abilities have been identified in other perceptual processes, such as visual *recognition—*the ability to identify visual objects. This ability is typically seen as a generalizable psychological process with substantial natural individual variation. For example, people who are better at recognizing some visual objects (e.g., faces) are also better at recognizing other visual objects (e.g., cars; Geskin & Behrmann, 2018; Richler et al., 2019). However, can the same be said of visual *comparison?* Does someone’s ability to “match” visual stimuli in one domain (e.g., faces) predict comparison performance in other domains (e.g., fingerprints)?

The current paper presents two experiments that are the first to explore whether there is a generalizable and domain-general psychological ability underpinning the ability to compare different complex visual stimuli, or whether these require separate skills. We explore individual differences in four visual comparison tasks to investigate the overlap or independence of performance in each task: face comparison, fingerprint comparison, firearms comparison, and a novel artificial print comparison task. Importantly, these tasks vary in familiarity—from familiar (faces) to unfamiliar (fingerprints and firearms) to entirely novel (artificial prints)—to ensure that accurate performance cannot be attributed to prior experience. If there is a generalizable ability underpinning visual comparison performance, we would expect performance in all comparison tasks to account for a substantial portion of shared variance across tasks. Conversely, if these are separate processes, we would expect performance in each comparison task to account for largely independent portions of variance. We also explore two alternative hypotheses: that individual differences in visual comparison accuracy are driven by intrinsic motivation as high performance could be determined by someone’s motivation to succeed (Experiment 1); or that individual differences in accuracy are driven by a broader visual-perceptual skill (Experiment 2). To examine this, participants in Experiment 1 also completed a measure of intrinsic motivation (the Intrinsic Motivation Inventory; McAuley et al., 1989; Tsigilis & Theodosiou, 2003) to determine whether any overlapping visual comparison ability is predicted by individual differences in motivation. In Experiment 2, participants also completed two other noncomparison visual-perceptual tasks (visual search and visual statistical learning) to determine whether the shared variance can be linked to a broader visual-perceptual ability.

## Experiment 1

### Method

#### Design

**Fig. 1:**
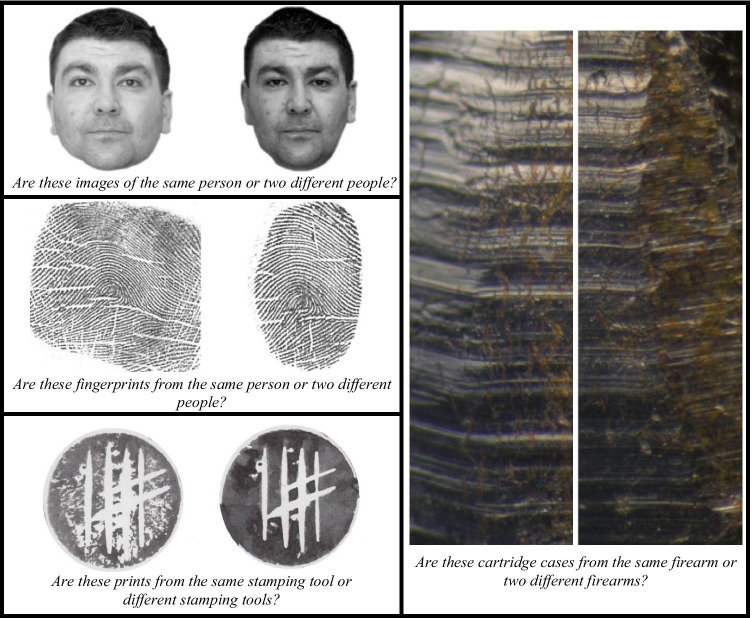
Example “match” trials for each comparison task (face: upper-left panel; fingerprint: middle-left panel; potato print; lower-left panel; firearms: right panel)

We used a within-subjects design where participants completed four comparison tasks (described below; see Fig. 1) and a measure of intrinsic motivation (the Intrinsic Motivation Inventory; McAuley et al., 1989; Tsigilis & Theodosiou, 2003) as a discriminant validity measure. The study preregistration data and analysis scripts can be found at https://osf.io/bvzpd/. Images used in this study are available upon request.

#### Participants

We recruited 124 participants online via Prolific Academic based on an a priori power analysis for detecting a two-sided correlation (*r* = .3) with 90% power (including an additional 10% to account for attrition). To be eligible for the study, participants were required to have normal or corrected-to-normal vision, live in the United States, have a Prolific approval rating of at least 95%, and have completed the experiment on a tablet or computer (not a mobile phone). No participants were excluded from the final sample as no participants met our preregistered exclusion criteria, which required them to correctly respond on at least three out of the five attention-checks (5.65% correctly passed just four questions; 94.35% passed all five).

Participants were 32.1 years old on average (*SD* = 12.1, range: 18–73), and the majority (62.9%) self-identified as female (36.3% male; 0.8% gender diverse) and White (64.5%; 13.7% Asian, 8.9% Black, 6.5% Hispanic, 6.5% Biracial, 0.81% Indian). Each participant was compensated USD$5.96 for completing the 50-minute experiment.

#### Tasks

Participants completed each of the four comparison tasks below. We selected two existing face and fingerprint comparison tasks (with minor modifications; Burton et al., 2010; Tangen et al., 2011), and created two additional novel comparison tasks: a cartridge case comparison task in firearms analysis, and a novel artificial-print comparison task. Pilot testing ensured each novel tests’ internal reliability and consistency were suitable for the assessment of individual differences (Siegelman et al., 2017; see Supplementary Materials on OSF). In cases where Cronbach’s α fell below recommended values for standardized tests (α > .8; Streiner, 2003a, 2003b) for our piloted tasks, we removed selected trials until α was ≥ .8.

##### Face comparison

Participants completed 40 face comparison trials (20 match and 20 nonmatch) from the Glasgow Face-Matching Task (GFMT-short; Burton et al., 2010; see upper-left panel of Fig. 1). The GFMT is a standardized face comparison task (Burton et al., 2010). Participants viewed two faces side by side and were asked, “Are these images of the same person or two different people?” on each trial. They responded by selecting one of two buttons (“same” or “different”) at the bottom of the screen.

##### Fingerprint comparison

Participants completed 56 fingerprint comparison trials (32 match and 32 nonmatch; see middle-left panel of Fig. 1) from the Fingerprint Matching Test from Tangen et al. (2011; Thompson & Tangen, 2014). Participants viewed two fingerprints side by side and were asked, “Are these fingerprints from the same person or two different people?” on each trial. They responded by selecting one of two buttons (“same” or “different”) at the bottom of the screen.

##### Firearms comparison

Participants completed 98 firearms comparison trials (49 match and 49 nonmatch; no trials were removed after pilot testing as α was ≥ .8) that were created for this experiment (see right panel of Fig. 1). Participants viewed two cartridge cases side by side and were asked, “Are these cartridge cases from the same firearm or two different firearms?” on each trial. They responded by selecting one of two buttons (“same” or “different”) at the bottom of the screen.

##### Artificial-print comparison

Participants completed 94 artificial-print comparison trials (47 match and 47 nonmatch; after excluding 10 trials based on pilot testing so that α ≥ .8) that were created for this experiment (see lower-left panel of Fig. 1). Artificial prints were created by carving the same basic pattern (four vertical lines and two diagonal intersecting lines inside a standardized circle) into potato halves. We then inked and stamped each half onto cardboard, dried the stamps, then scanned and digitized all prints.

Participants viewed two artificial prints side by side and were asked, :Are these prints from the same stamping tool or two different stamping tools?” on each trial. They responded by selecting one of two buttons (“same” or “different”) at the bottom of the screen.

##### Intrinsic motivation inventory

Participants completed a measure of their intrinsic motivation and subjective experience during the experiment: the Intrinsic Motivation Inventory (McAuley et al., 1989). The Intrinsic Motivation Inventory is a validated measure of intrinsic motivation as it has acceptable reliability and stability (McAuley et al., 1989; Tsigilis & Theodosiou, 2003) and has been used across multiple domains—from education to mental health research (Choi et al., 2010; Leng et al., 2010; Monteiro et al., 2015).

Participants completed three subscales of the inventory: the Effort, Enjoyment, and Perceived Competence subscales. They answered questions on a 7-point Likert scale from *not at all true* to *very true*. They answered questions such as, “I put a lot of effort into this” (effort subscale); “I enjoyed doing this activity very much” (enjoyment subscale); and “I am satisfied with my performance in this task” (perceived competence subscale). A full list of the questions can be found at https://selfdeterminationtheory.org/intrinsic-motivation-inventory/.

#### Dependent measures

Comparison performance in each task was computed using the signal-detection measure sensitivity (*d'*; Phillips et al., 2001; Stanislaw & Todorov, 1999). Higher *d'* values indicate higher sensitivity to the presence of a target stimulus independent of a tendency to respond “same” or “different” (response bias) and higher values are typically interpreted as higher “accuracy” in a task. We also calculated participants’ criterion (C)—a measure of tendency to respond ‘same’ or different—in each task and these analyses can be found in the Supplementary Materials on OSF (https://osf.io/bvzpd/).

Intrinsic motivation scores were calculated by averaging participants’ Likert-scale responses on the Effort, Enjoyment, and Perceived Competence inventory subscales (including the reverse-scored items).

#### Procedure

Participants completed the experiment via an online survey platform Qualtrics (https://www.qualtrics.com/). Participants completed all four comparison tasks in a randomized order, and all trials within each comparison task in a pseudo-randomized order (where one trial order was randomly generated when coding the experiment in each task and all participants completed trials in this order) to minimize error variance (Mollon et al., 2017). At the beginning of each comparison task, participants received brief task instructions and completed two practice trials where they were given corrective feedback (one match and one nonmatch). Upon completion of the comparison tasks, participants then completed the three subscales of the intrinsic motivation inventory, provided demographic information, and then viewed a debriefing statement.

### Results and discussion

#### Descriptive results and psychometrics

The descriptive statistics and psychometric properties of all five tasks are presented in Table 1. Sensitivity was significantly above chance (i.e., above 0) on all four comparison tasks—face: *t*(123) = 27.23, *p* < .001; fingerprint: *t*(123) = 19.90, *p* < .001; firearms: *t*(123) = 33.43, *p* < .001; artificial-print: *t*(123) = 21.56, *p* < .001. Psychometric properties for all five measures were close to or above recommended values for standardized tests on a typical measure of scale reliability (see Table 1; Cronbach's α > .8; Streiner, 2003a, 2003b), except for the fingerprint comparison task where the values fell below typically recommended values for test evaluation (α = .61).

**Table 1:** Descriptive statistics for each task (standard deviation in parentheses) Task performance for face, fingerprint, firearms, and artificial prints are shown in *d'*, while intrinsic motivation is the mean response rating

	Mean task performance	α	Skewness	Kurtosis
Face comparison	2.21 (.90)	.75	−.10	2.59
Fingerprint comparison	1.06 (.59)	.61	.11	3.04
Firearms comparison	2.90 (.97)	.92	−1.10	3.80
Artificial-print comparison	1.21 (.63)	.82	−.01	3.28
Intrinsic motivation	4.88 (1.06)	.94	.16	2.40

Task performance for face, fingerprint, firearms, and artificial prints are shown in *d'*, while intrinsic motivation is the mean response rating. Cronbach’s alpha was calculated on raw accuracy scores per participant (not *d'* scores)

#### Correlations between comparison performance

**Fig. 2:**
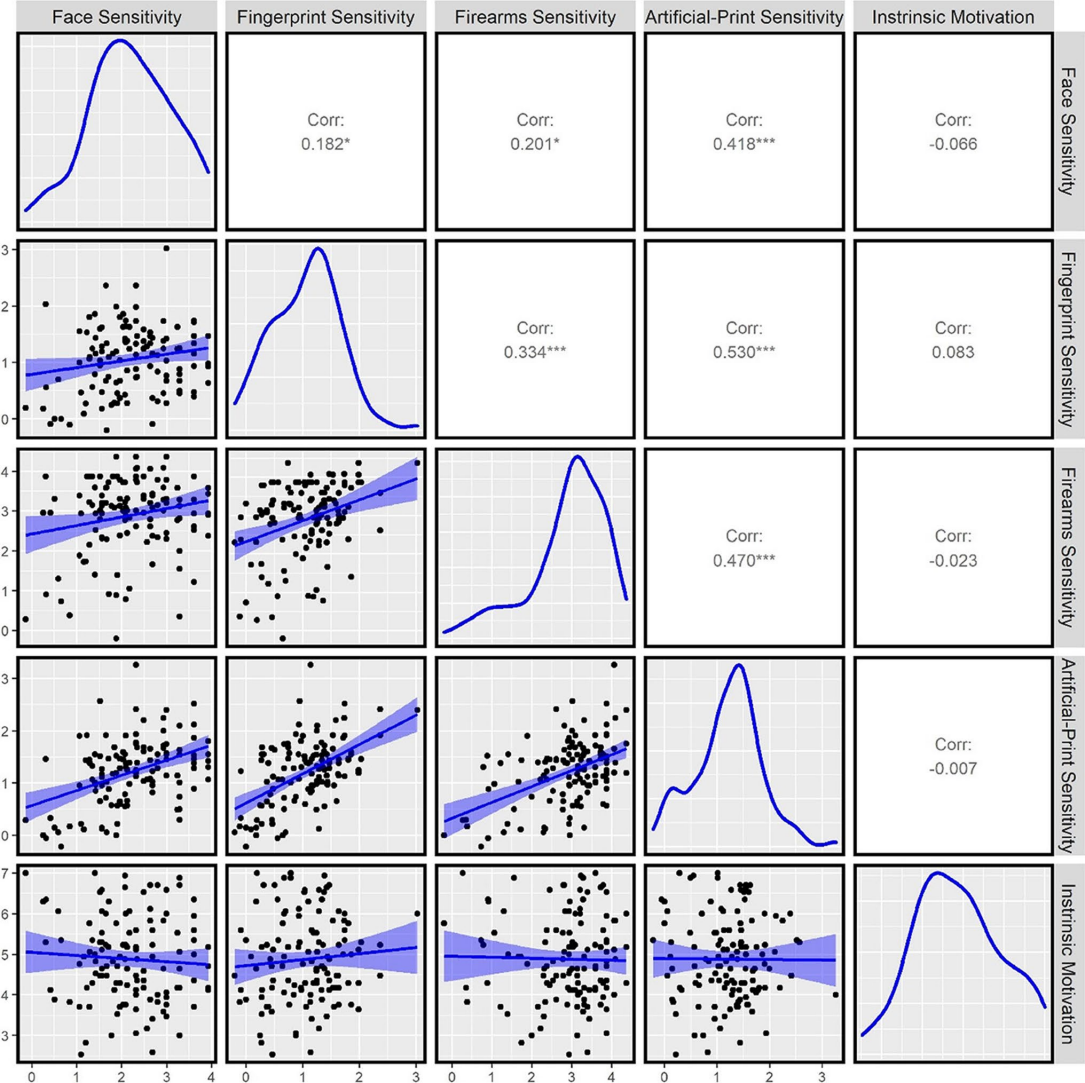
Pearson correlations between task performance in Experiment 1

To investigate the relationships between task performance, we calculated Pearson’s correlations between sensitivity on all comparison tasks. Sensitivity on all comparison tasks was significantly and positively correlated with one another (see Fig. 2, and Table 5 in the Appendix for detailed statistics).^1^ We also calculated Bayes Factors to examine the likelihood of the observed data under the null hypothesis (i.e., the absence of correlations) compared with an alternative hypothesis (i.e., the presence of correlations) using the BayesFactor package in R (Morey et al., 2018). We observed a Bayes Factor >10 supporting the alternative hypothesis for four of the six comparison sensitivity correlations, providing strong evidence for the observed positive correlations (Wetzels et al., 2011). Smaller Bayes factors were observed for the remaining two sensitivity correlations (face and fingerprint: *BF* = 1.47, face and firearms: *BF* = 2.26)—providing weaker support for the presence of correlations between these tasks (see Table 5 in the Appendix).

#### Correlations between comparison performance and intrinsic motivation

**Fig. 3:**
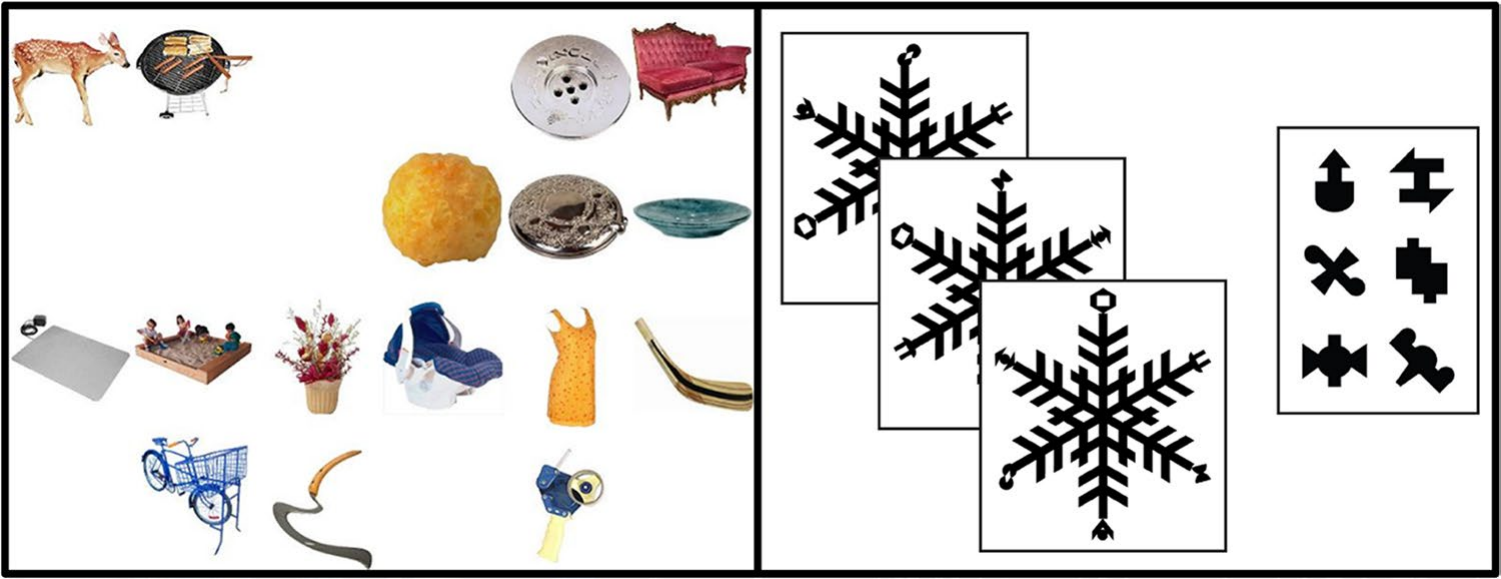
Two discriminant validity tasks used in Experiment 2: Visual search (left panel) and visual statistical learning (right panel)

To investigate the relationship between each comparison task and intrinsic motivation, we calculated Pearson’s correlations between intrinsic motivation and sensitivity separately. Importantly, intrinsic motivation did not significantly correlate with sensitivity on any comparison tasks (see Table 5, in the Appendix, and Fig. 3). We observed a Bayes factor of less than or close to .3 for all correlations between intrinsic motivation and sensitivity in each comparison task, which provides substantial evidence for the absence of correlations (Wetzels et al., 2011).

#### Principal component analysis (PCA)

We explored the shared and unshared variance in sensitivity values across the four comparison tasks and intrinsic motivation scores with a Principal Component Analysis (PCA) using the *prcomp* function from the core *stats* package in R. Rotation was not conducted in the PCA. The loadings of all tasks on the five components and the proportion of variance explained by each component can be seen in Table 2.

**Table 2:** Results of the principal components analysis (loadings matrix and percentage of variance explained)

	Component 1	Component 2	Component 3	Component 4	Component 5
Face comparison	.40	−.32	.80	.08	−.32
Fingerprint comparison	.50	.27	−.26	−.64	−.45
Firearms comparison	.48	−.00	−.42	.73	−.26
Artificial-print comparison	.59	−.01	.02	−.10	.79
Intrinsic motivation	<.01	.91	.36	.21	.02
*Variance explained*	*41.99%*	*20.95%*	*16.37%*	*13.10%*	*7.60%*

Component 1 explained a substantial portion of the variance across all five tasks (41.99%), and sensitivity on all four comparison tasks loaded strongly onto this component, but intrinsic motivation did not. This suggests that this component represents a generalizable comparison ability unrelated to intrinsic motivation. Component 2 explained an important portion of the variance across all tasks (20.95%) and intrinsic motivation scores loaded strongly onto this component, with sensitivity in each comparison task loading weakly or not at all onto this component. This suggests intrinsic motivation represents separate and unshared variance to performance on all comparison tasks.

Components 3–5 also explained an important portion of the variance across all tasks (37.07%; from 16.37–7.60%). Face comparison sensitivity strongly loaded onto Component 3 alone which explained the next greatest portion of variance (16.37%), fingerprint and firearms comparison sensitivity strongly loaded on Component 4 (positively correlated with firearms comparison and negatively correlated with fingerprint comparison; explaining 13.10% of variance), while artificial-print comparison sensitivity strongly loaded onto Component 5 alone (explaining 7.60% of variance). Overall, these results suggested that sensitivity on all comparison tasks reflect a mixture of shared (Component 1) and nonshared variance (Components 3, 4, and 5), whilst intrinsic motivation scores reflect separate nonshared variance (Component 2).

Experiment 1 explored whether there is a generalizable, domain-general perceptual skill underlying the comparison of visual stimuli and whether this relationship could be attributed to intrinsic motivation. Participants’ visual comparison sensitivity significantly correlated on all four tasks and accounted for a substantial portion of the variance in performance across all tasks—but intrinsic motivation did not and accounted for a separate portion of the variance. These results provide the first indication of a domain-general visual comparison ability that varies naturally in the general population.

## Experiment 2

While the results of Experiment 1 suggest there is shared ability across visual comparison performance, these results may reflect a broader perceptual visual ability—rather than a skill specific to visual *comparison*. To investigate this possibility, Experiment 2 examined whether there is a relationship between visual comparison performance and performance on two other tasks that rely on visual-perceptual skills: visual search and visual statistical learning.

Visual search tasks are measures of attentional deployment and control that ask participants to search for a target among surrounding distractors (for review, see Chan & Hayward, 2013). Visual statistical learning is the ability to extract and encode statistical information from the visual environment around you (e.g., learning that black or white cars are more common than yellow cars; Fiser & Aslin, 2001; Turk-Browne et al., 2005). We selected these two tasks as they both engage processing of visual-perceptual information and show stable individual differences (e.g., visual search: Ericson et al., 2017; and, e.g., visual statistical learning: Growns et al., 2020). Importantly however, these tasks are theoretically unrelated to the ability to *compare* and evaluate similarity between visual stimuli. Therefore, we predict that if there is a domain-general ability specific to visual comparison, performance across visual comparison tasks will correlate and load similarly onto the same component in the PCA, but visual search and visual statistical learning performance will not.

### Method

#### Design

We used a within-subjects design where participants completed six tasks: face comparison, fingerprint comparison, firearms comparison, artificial-print comparison, visual search, and visual statistical learning. The study preregistration, data and analysis scripts can be found at https://osf.io/bvzpd/.

#### Participants

We recruited 124 participants online via Prolific Academic informed by the same power analysis as in Experiment 1. To be eligible for the study, participants were required to have normal or corrected-to-normal vision, live in the United States, have a Prolific approval rating of at least 95%, and have completed the experiment on a tablet or computer (not a mobile phone). All participants passed our preregistered exclusion criteria which required them to correctly respond to at least three of five attention-checks to be included in the final sample (0.81% correctly passed just three questions, 21.77% passed just four questions, and 77.42% just passed all five).

Participants were 29.3 years old on average (*SD* = 11.2, range: 18–66), and the majority (62.1%) self-identified as male (37.1% female; 0.8% gender diverse) and White (77.4%; 9.7% Asian, 6.5% Black, 3.2% Hispanic, 3.2% Other). Each participant was compensated £5.85 for completing the 70-minute experiment.

#### Tasks and dependent measures

Participants completed four visual comparison tasks: face comparison, fingerprint comparison, firearms comparison, and artificial-print comparison). The firearms and artificial-print tasks from Experiment 1 were retained, but participants completed two new face and fingerprint comparison tasks (described further below). Performance in visual comparison tasks was measured by calculating a signal detection measure of sensitivity as in Experiment 1. Participants also completed two additional tasks as measures of discriminant validity which were both pilot-tested to ensure they were reliable and variable enough to appropriately measure individual differences: visual search and visual statistical learning.

##### Face comparison

Participants completed 80 face comparison trials (40 match and 40 nonmatch) from the Glasgow Face Matching Task 2—Short Form (GFMT2-S; White et al., 2021). The GFMT2-S is an updated version of the GFMT that was created to be more difficult and representative of real-world face identification tasks (i.e., variation in head angle, pose, expression, and image quality) than the original task. Participants were asked to answer the same question (“Are these images of the same person or two different people?”) as in Experiment 1 and responded by selecting one of two buttons (“same” or “different”) at the bottom of the screen.

##### Fingerprint comparison

Participants completed 40 fingerprint comparison trials (20 match and 20 nonmatch) which were a subset from the fingerprint task in Growns and Kukucka (2021). This subset was chosen to be most representative of fingerprint comparison skill using the same method to select trials in the GFMT-2: item-to-test correlations were calculated for each trial from pilot data for Growns and Kukucka (2021) and the 20 match and 20 nonmatch trials with the highest correlations were then selected. Participants were asked to answer the same question (“Are these fingerprints from the same person or two different people?”) as in Experiment 1 and responded by selecting one of two buttons (“same” or “different”) at the bottom of the screen.

##### Visual search

Participants completed 120 trials in a visual search task developed for use in this experiment (10 blocks of 12 trials + 1 practice block with 12 trials; see left panel of Fig. 3). On each trial, participants were instructed to indicate if a target object (e.g., the deer in Fig. 3) was present or absent in an array of 16 objects by pressing “P” on the keyboard if the target was present or “A” if the target was absent in the array. The target object was present on 50% of trials in each block and each block had a different target object. Performance on this task was measured by calculating the mean reaction time (RT) on correct target-present trials only (Cunningham & Wolfe, 2012; Wolfe, 2012). Shorter reaction times indicate better visual search performance.

##### Visual statistical learning

Participants completed a visual statistical learning task adapted from previous research (Growns et al., 2020; Growns & Martire, 2020a) where participants first completed an exposure phase and then a test phase. During the exposure phase, participants viewed 60 complex patterns (see right panel of Fig. 3) in a randomized order (each pattern displayed for 3-sec with a 200-ms interval in-between) and were instructed to pay attention to them as they would be asked some questions about them afterwards. Each pattern contained different features (see right images in the right panel of Fig. 3) on the ends of the pattern ‘arms’ that occurred with different statistical frequencies across all patterns (e.g., feature “A” appeared in 10% of patterns, whilst feature “B” appeared in 20% of patterns).

During the test phase, participants completed 45 trials where they were tested on how well they learned the frequencies, by being asked which of 2, 3, or 4 features were more familiar to them. Performance on this task was measured by calculating the number of trials participants correctly chose the most frequent feature, where higher scores indicated better statistical learning. Chance performance on this task was 16.62 trials (36.9% accuracy).

#### Procedure

Participants completed the experiment via an online testing platform (Testable.org) that better captures reaction time data than other online platforms (e.g., Qualtrics; de Leeuw & Motz, 2016; Rezlescu et al., 2020). Participants first provided demographic information and then completed all six tasks in a randomized order and completed all trials in each task in a randomized order. Note this differs from Experiment 1 where participants completed tasks in a randomized order but completed trials within each task in a pseudo-randomized order. At the beginning of each task, participants received brief task instructions and completed two practice trials where they were given corrective feedback. Upon completion of all tasks, participants viewed a debriefing statement.

### Results and discussion

#### Descriptive results and psychometrics

The descriptive statistics and psychometric properties of all six tasks are presented in Table 1. Sensitivity was significantly above chance (i.e., above 0) on all four comparison tasks—face: *t*(123) = 30.51, *p* < .001; fingerprint: *t*(123) = 9.86, *p* < .001; firearms: *t*(123) = 35.84, *p* < .001; artificial-print: *t*(123) = 21.68, *p* < .001. Visual search performance was within the range seen within previous experiments (Cunningham & Wolfe, 2012; Wolfe, 2012), and statistical learning performance was also significantly above chance (i.e., above 36.9%), *t*(123) = 12.63, *p* < .001. Psychometric properties for all six measures were close to or above recommended values for standardized tests on a typical measure of scale reliability (see Table 3; Cronbach's α > .8; Streiner, 2003a, 2003b), except for the fingerprint comparison task (α = .62).

**Table 3:** Descriptive statistics for each task (standard deviation in parentheses)

	Task performance	α	Skewness	Kurtosis
Face comparison	1.97 (0.72)	.79	.24	3.30
Fingerprint comparison	.58 (0.66)	.62	−.21	3.36
Firearms comparison	2.81 (0.88)	.90	−.76	3.49
Artificial-print comparison	1.00 (0.51)	.74	−.30	3.10
Visual search	1002 (636.57)	.86	.65	4.22
Visual statistical learning	58.15% (18.74)	.88	.39	2.24

Task performance for face, fingerprint, firearms, and artificial prints are shown in *d’*, visual search is mean reaction time (ms) on correct target-present trials, and visual statistical learning is percentage correct. Cronbach’s alpha was calculated on raw accuracy scores per participant for all tasks

#### Correlations between visual comparison performance

**Fig. 4:**
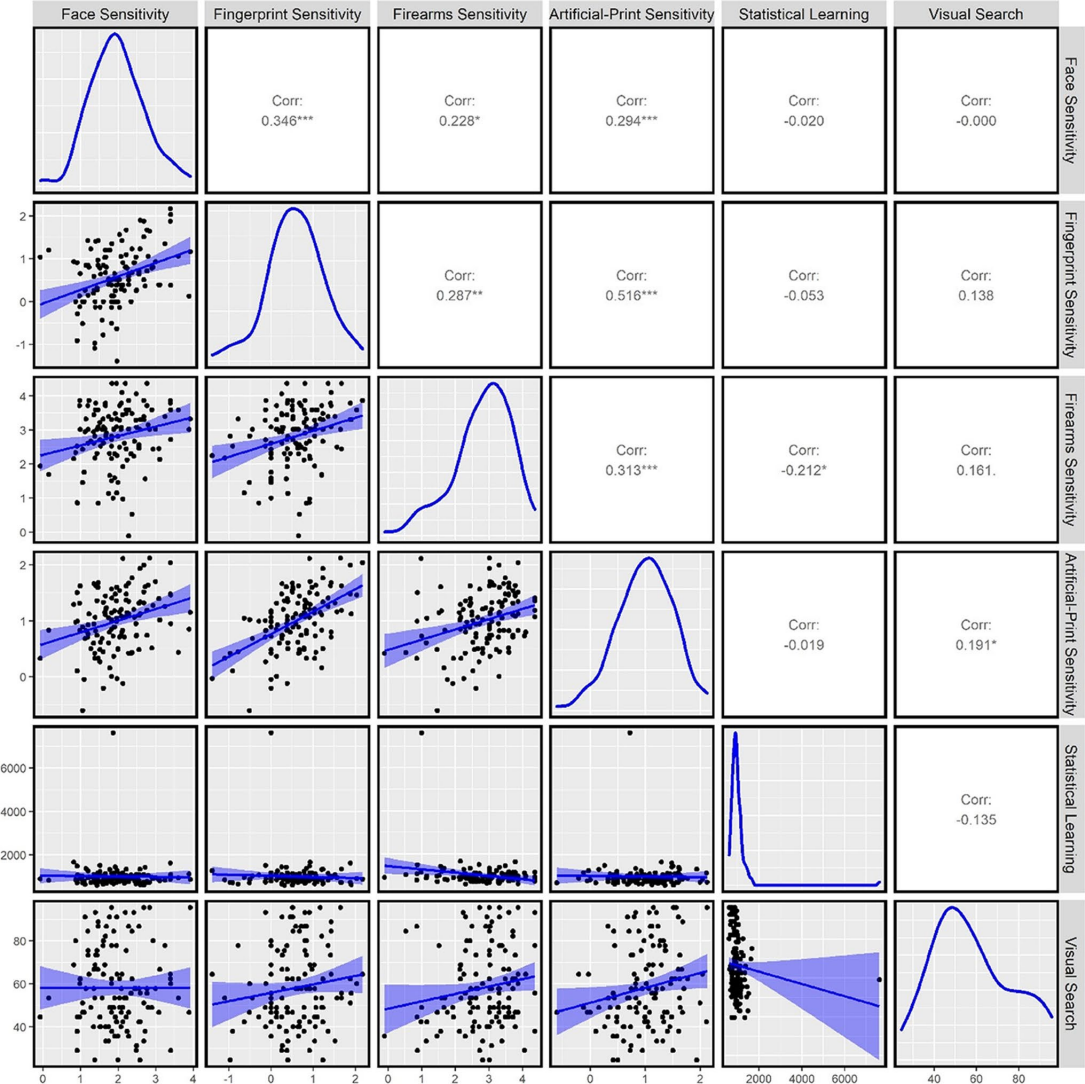
Pearson correlations between task performance in Experiment 2

To investigate the relationship between visual comparison tasks, we calculated Pearson’s correlations between each. Sensitivity on all comparison tasks was significantly and positively correlated with one another (see Fig. 4, and Table 6 in the Appendix). We also observed Bayes Factors >10 for five of the six comparison sensitivity correlations which provides strong support for the presence of all correlations, with weaker support for the presence of a correlation between face and firearms comparison (*BF* = 4.57).

#### Correlations between visual comparison performance, visual search, and visual statistical learning

To investigate the relationship between each comparison task, visual search, and visual statistical learning performance, we calculated Pearson’s correlations (see Fig. 4, and Table 6 in the Appendix). Neither visual search nor statistical learning performance significantly correlated with comparison sensitivity on any task—except for correlations between visual search and firearms sensitivity (*r* = .212, *p* = .018) and between statistical learning and artificial-print sensitivity (*r* = .191, *p* = .033). Yet it is important to note that the correlation between visual search and firearms sensitivity is likely spurious and driven by an outlier (see Fig. 4). Visual search and visual statistical learning were also not significantly correlated with each other (*r* = −.135, *p* = .136). It is important to note that these correlations were weaker than any of the correlations between visual comparison tasks. We also calculated correlations between log-transformed reaction-time data and the pattern of correlations was consistent (see Supplementary Materials on OSF; except for the significant correlation between visual search performance and artificial-print sensitivity which was no longer significant).

Based on Bayesian analysis, there was substantial support (*BF* <.33) for the *absence* of correlations between four of the eight correlations between comparison performance and visual search/statistical learning, anecdotal support (*BF* >.33 & <1.0) for the absence of two of the eight correlations, and anecdotal support for the presence of the correlations between visual search/firearms sensitivity and statistical learning/artificial-print sensitivity.

#### Principal component analysis (PCA)

We explored the shared and unshared variance in sensitivity values across the four comparison tasks and two discriminant validity tasks with a Principal Component Analysis (PCA) using the *prcomp* function from the core *stats* package in R. Rotation was not conducted in the PCA. The loadings of all tasks on the six components and the proportion of variance explained by each component can be seen in Table 4.

**Table 4:** Results of the principal components analysis (loadings matrix and percentage of variance explained)

	Component 1	Component 2	Component 3	Component 4	Component 5	Component 6
Face comparison	.40	−.35	.35	−.63	−.42	−.10
Fingerprint comparison	.53	−.21	−.08	.00	.44	.69
Firearms comparison	.44	.25	.26	.62	−.53	.11
Artificial-print comparison	.53	−.17	−.23	.19	.33	−.70
Visual search	−.16	−.69	−.51	.25	−.40	.10
Visual statistical learning	.24	.51	−.70	−.34	−.28	.07
*Variance explained*	*34.92%*	*19.07%*	*15.35%*	*11.55%*	*11.21%*	*7.89%*

Component 1 explained a substantial portion of the variance across all six tasks (34.92%) and sensitivity on all four comparison tasks loaded strongly onto this component, but visual search and visual statistical learning did not. This suggests that this component represents a generalizable comparison ability unrelated to performance on the other two tasks. Component 2 explained an important portion of the variance across all tasks (19.07%) and both visual search and visual statistical learning loaded strongly onto this component, while sensitivity on each comparison task loaded weakly. As lower visual search scores indicate better performance, this pattern of results suggests that high statistical learning performance is associated with high visual search performance. Together, this pattern suggests that visual comparison performance is explained by a shared factor that is independent of the other visual-perceptual tasks and demonstrates discriminant validity between these two constructs.

Components 3–6 also explained an important portion of the variance across all tasks (43.01%; from 15.35%–7.89%). Visual search and statistical learning strongly loaded onto Component 3 alone which explained the next greatest portion of variance (15.35%; negatively correlated with both), and face comparison and firearms comparison sensitivity strongly loaded onto Component 4 (negatively correlated with face comparison and positively correlated with firearms sensitivity; explaining 11.55% of variance). Firearms comparison, face comparison, fingerprint comparison, and visual search strongly loaded onto Component 5 (positively correlated with fingerprint comparison and negatively correlated with firearms, face, and visual search; explaining 11.21% of variance), and fingerprint and artificial-print comparison sensitivity strongly loaded onto Component 6 (positively correlated with fingerprint comparison and negatively correlated with artificial-print comparison; explaining 7.89% of variance). Overall, these results suggested that sensitivity on all comparison tasks reflects a mixture of shared (Component 1) and nonshared variance (Components 3, 4, 5, and 6), whilst visual search and visual statistical learning reflect separate nonshared variance (Components 2 and 3).

Experiment 2 explored whether individual differences in visual performance could be accounted for by broader perceptual skill in visual tasks. Consistent with Experiment 1, participants’ visual comparison sensitivity significantly correlated on all four comparison tasks and accounted for a substantial portion of the variance in performance across all tasks—but visual search and visual statistical learning performance did not and accounted for a separate portion of variance (except for correlations between firearms/visual search and artificial-print comparison/statistical learning which were significant but weaker than all visual comparison correlations). These results provide further evidence that there is an underlying generalizable ability for comparing visual stimuli that is largely unrelated to other visual-perceptual tasks.

## General discussion

Across two experiments, we explored whether there is a generalizable and domain-general perceptual skill underpinning the ability to compare—or “match”—different visual stimuli. Participants’ sensitivity in four different comparison tasks were all significantly correlated with each other, and a substantial portion of variance (41.99% in Experiment 1 and 34.92% in Experiment 2) across all tasks was accounted for by one shared “matching” component in both experiments. Together, these results support the conclusion that individual differences in visual comparison accuracy are explained by a shared ability that generalizes across a range of visual stimuli. Notably, intrinsic motivation (Experiment 1), visual search and visual statistical learning (Experiment 2) did not significantly correlate with sensitivity in any comparison task and loaded onto separate components that accounted for large proportions of the variance across all tasks (20.95% in Experiment 1 and 19.07% in Experiment 2). This suggests that individual differences in visual comparison cannot be attributed to individual differences in intrinsic motivation or other visual-perceptual tasks.

Importantly, our study also provides evidence of stimulus-specific individual differences. This is reflected in the moderate correlations seen between sensitivity in all comparison tasks across both experiments, and the principal components analysis, where additional components featured loadings from just one or a subset of comparison tasks. This suggests there are also likely individual stimulus-specific skills where some people are better at comparing specific stimuli over other stimuli. Overall, our results are the first to suggest that visual comparison is an interplay between an overarching generalizable comparison ability, as well as individual stimulus-specific ability.

This stimulus-specific skill may be partially attributed to stimulus familiarity and experience. Face-comparison performance—the most familiar stimuli—demonstrated the highest stimulus-specific variance: face-comparison sensitivity had the lowest average correlation with all other tasks (*r* = .267 in Experiment 1 and .289 in Experiment 2); and accounted for the third to fourth-largest portion of variation (16.37% in Experiment 1 and 11.55% in Experiment 2) across all tasks. In contrast to faces, fingerprint, firearms and artificial-print sensitivity accounted for less variance in our data—where familiarity with these stimuli ranges from unfamiliar to entirely novel. This is consistent with research that suggests there is a shift from domain-general to domain-specific mechanisms with increased perceptual experience in a domain (Chang & Gauthier, 2020, 2021; Sunday et al., 2018; Wong et al., 2014; Wong & Gauthier, 2010, 2012), and research that links experience and visual comparison performance (Thompson & Tangen, 2014).

Our results highlight visual comparison as a natural and generalizable ability that varies in the general population—yet the precise mechanisms underpinning this skill are only beginning to be explored (see Growns & Martire, 2020b, for review). It is possible that holistic processing—or the ability to view images as a ‘whole’ rather than a collection of features (Maurer et al., 2002)—underpins visual comparison performance: both facial and fingerprint examiners show evidence of holistic processing when viewing domain-specific stimuli (Busey & Vanderkolk, 2005; Towler, White, & Kemp, 2017b; Vogelsang et al., 2017). In contrast, featural processing—or the ability to view images as separate features—is also important in visual comparison. Professional performance is improved when examiners have an opportunity to engage featural processing: both facial and fingerprint examiners demonstrate greater performance gains than novices in domain-specific visual comparison tasks (Thompson et al., 2014; Towler, White, & Kemp, 2017; White, Phillips, et al., 2015). Novices’ face-comparison performance also correlates with featural processing tasks such as the NAVON and figure-matching tasks (Burton et al., 2010; McCaffery et al., 2018), and novices’ comparison performance is improved by instructing participants to rate or label features (Searston & Tangen, 2017c; Towler, White, & Kemp, 2017b). Low-performing novices also derive greater benefit from featural comparison training than high-performers—suggesting high-performers may already use such strategies (Towler, Keshwa, et al., 2021b). The role of holistic and featural processing in visual comparison performance remains an important avenue for future research.

These results have important applied implications. Whilst empirically based training for existing examiners is important to improve ongoing professional performance (Growns & Martire, 2020a), our results suggest that larger gains in performance could be achieved by selecting trainee examiners based on visual comparison ability. A similar approach has been used in applied domains: recruiting individuals with superior face recognition improves performance in real-world face identification tasks (Robertson et al., 2016; White, Dunn, et al., 2015). Professional performance in other forensic feature-comparison disciplines could likely be similarly improved by recruiting individuals with superior performance on a test battery of visual comparison tasks. Importantly, our results do *not* suggest that examiners would benefit from practicing outside of their primary domain of experience. Despite identifying a generalizable visual comparison ability, we also identified individual differences in stimulus-specific skills that suggest part of accurate visual comparison performance *is* domain specific.

As the participants in this study were untrained novices, it is unclear whether these results could generalize to practicing professionals. While investigating individual differences in the general population requires a novice sample, it is entirely plausible that a domain-general visual comparison mechanism may be diminished or negated for experts in this task as expertise is typically conceptualized as narrow and domain-specific (Charness et al., 2005; Ericsson, 2007, 2014). However, emerging evidence suggests domain-specific expertise may lend advantages to domain-general skill. For example, although facial examiners outperform fingerprint examiners in face comparison (i.e., facial examiners’ domain-specific expertise), fingerprint examiners outperform novices in the same task—despite it being outside their primary area of expertise (Phillips et al., 2018). Whether this domain-general advantage is developed alongside domain-specific expertise or is the result of preexisting individual differences in this ability will be an important avenue for future research.

This study provided the first evidence of a generalizable ability to underpinning the ability to compare or “match” different, complex visual stimuli. We demonstrated that the ability to compare stimuli such as faces, fingerprints, firearms, and artificial prints is in part due to a generalizable and domain-general ability—although subject to stimulus-specific constraints. These results have important theoretical and applied implications for both behavioural and forensic science. Importantly, test batteries of visual comparison tasks could be used to identify and recruit top-performing individuals to improve performance in forensic feature-comparison disciplines.

## Appendix

**Table 5:** Correlations for performance between tasks in Experiment 1 (Pearson correlations reported with *p* values in parentheses, with Bayes factors displayed below)

	Face comparison	Fingerprint comparison	Firearms comparison	Artificial-print comparison
Face comparison	–			
Fingerprint comparison	.182 (.043) BF = 1.47	–		
Firearms comparison	.201 (.026) BF = 2.26	.334 (< .001) BF = 202.73	–	
Artificial-print comparison	.418 (<.001) BF = 1.50e4	.530 (<.001) BF = 4.47e7	.470 (<.001) BF = 4.30e5	–
Intrinsic motivation	−.066 (.468) BF = .27	.083 (.360) BF = .31	−.023 (.801) BF = .21	−.007 (.937) BF = .21

**Table 6:** Correlations for performance between tasks in Experiment 2 (Pearson correlations reported with *p* values in parentheses, with Bayes factors displayed below).

	Face comparison	Fingerprint comparison	Firearms comparison	Artificial-print comparison	Visual search
Face comparison	–				
Fingerprint comparison	.346 (<.001) BF = 346.33	–			
Firearms comparison	.228 (.011) BF = 4.57	.287 (.001) BF = 30.65	–		
Artificial-print comparison	.294 (<.001) BF = 39.56	.516 (<.001) BF = 1.37e7	.313 (<.001) BF = 83.84	–	
Visual search	−.020 (.823) BF = .21	−.053 (.562) BF = .24	−.212 (.018) BF = 3.04	−.019 (.835) BF = .21	–
Visual statistical learning	−.00 (.997) BF = .21	.138 (.126) BF = .63	.161 (.073) BF = .96	.191 (.033) BF = 1.80	−.135 (.136) BF = .60

## Footnotes

1 Note we also conducted these analyses with outliers (scores 2 *SD*+ above the mean) and negative performers (scores below zero) removed and the pattern of results was consistent (see Supplementary Materials on OSF: https://osf.io/bvzpd/), except for two face comparison correlations when negative performers were removed which may be due to face comparison having the highest stimulus-specific variance.
